# The survival benefit of increasing the number of active drugs for metastatic colorectal cancer: A multicenter retrospective study

**DOI:** 10.1002/cam4.4599

**Published:** 2022-02-19

**Authors:** Takeshi Kawakami, Toshiki Masuishi, Yasuyuki Kawamoto, Hirofumi Go, Kyoko Kato, Ryosuke Kumanishi, Kentaro Sawada, Satoshi Yuki, Kouji Yamamoto, Yoshito Komatsu, Kei Muro, Kunihiro Fushiki, Hiromichi Shirasu, Kentaro Yamazaki

**Affiliations:** ^1^ Department of Gastrointestinal Oncology Shizuoka Cancer Center Shizuoka Japan; ^2^ Department of Clinical Oncology Aichi Cancer Center Hospital Nagoya Japan; ^3^ Division of Cancer Center Hokkaido University Hospital Sapporo Japan; ^4^ Department of Biostatistics Yokohama City University Yokohama Japan; ^5^ Department of Gastroenterology and Hepatology Hokkaido University Hospital Sapporo Japan; ^6^ Department of Medical Oncology Kushiro Rosai Hospital Kushiro Japan

**Keywords:** colorectal cancer, continuum of care, drug availability, late‐line treatment, regorafenib, trifluridine/tipiracil

## Abstract

**Background:**

The development of chemotherapy and treatment strategies for metastatic colorectal cancer (mCRC) have provided patients with significant survival benefits. Currently, molecular targeting agents and late‐line treatment with regorafenib and trifluridine/tipiracil (FTD/TPI) are available. However, the impact of this increase in drug availability on overall survival (OS) in mCRC remains a clinical question.

**Methods:**

We retrospectively collected data on consecutive mCRC patients who were treated at three institutions in Japan. We divided the patients into three cohorts: patients who initiated first‐line treatment from Jan 2005 to Dec 2006 (cohort A: only cytotoxic drugs available), Jan 2007 to Dec 2011 (cohort B: molecular targeting drugs available), and Jan 2012 to Sep 2016 (cohort C: late‐line treatment available).

**Results:**

A total of 1409 consecutive patients were analyzed. The median survival time (MST) in cohorts A, B, and C was 18.6, 25.4, and 26.4 months, respectively. The hazard ratio (HR) for cohort B versus A was 0.81 (95% CI 0.68–0.97), for cohort C versus A was 0.74 (95% CI 0.61–0.89), and for cohort C versus B was 0.92 (0.81–1.03). The median number of administered drugs (range) was 3 (1–5) in cohort A, 4 (1–7) in cohort B, and 4 (1–7) in cohort C. The increase in drug availability extended the MST from 15.5 months in patients treated with ≤3 drugs to 36.0–37.3 months in patients treated with six to seven drugs.

**Conclusion:**

The development of chemotherapy including late‐line treatments could improve the prognosis of mCRC patients.

## Graphical abstract

This multicenter retrospective study investigated whether a strategy of administering all available drugs leads to a better prognosis in metastatic colorectal cancer.

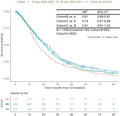

## 1 BACKGROUND

Colorectal cancer is the third most common cancer, and 1.9 million cases were newly diagnosed in 2020. Colorectal cancer is the second leading cause of cancer death worldwide.^1^ In Japan, colorectal cancer was the most common cancer in 2017 and was the second most prevalent cause of death in 2019.^2^ The prognosis of metastatic colorectal cancer (mCRC) is poor: the overall survival (OS) of patients without any treatment is 4–6 months.^3^ After 5‐fluorouracil (5‐FU)/leucovorin (LV) showed a survival benefit for mCRC against the best supportive care in the late 1980s,^4^, ^5^, ^6^ irinotecan or oxaliplatin‐based combination therapy was introduced in the late 1990s.^7^, ^8^, ^9^ Furthermore, combination therapy with molecular targeting agents such as bevacizumab or anti‐EGFR antibody with cytotoxic drugs was introduced in the 2000s.^10^, ^11^, ^12^, ^13^, ^14^ These intensive combination therapies not only prolong survival, but also enhance tumor shrinkage and increase the chances of conversion surgery. Additionally, treatment with regorafenib or trifluridine/tipiracil (FTD/TPI) focusing on late‐line treatment, which is a clinical unmet need, was developed in the 2010s.^15^, ^16^ Recently, personalized treatments for mCRC have progressed with the spread of biomarker testing for genes such as *RAS, BRAF* ^V600E^ mutation, and microsatellite instability.

While recent clinical trials of first‐line treatment for mCRC have been able to extend the OS to over 30 months,^17^ the median progression‐free survival in most clinical trials has consistently been around 10 months.^13^, ^18^, ^19^ Therefore, subsequent treatments after first‐line chemotherapy including regorafenib or FTD/TPI as a late‐line treatment may prolong OS. There are few studies on the extent to which these subsequent treatments improve survival over the entire treatment period. In the present study, we investigated the impact of the increase in drug availability on the OS of mCRC from the initiation of first‐line treatment using real‐world data.

## 2 PATIENTS AND METHODS

mCRC patients who received first‐line treatment between January 2005 and November 2016 at three institutions (Shizuoka Cancer Center, Aichi Cancer Center Hospital, and Hokkaido University Hospital) were selected. Inclusion criteria were as follows: (1) pathologically confirmed adenocarcinoma of the colon or rectum, (2) age over 20 years at the initiation of first‐line chemotherapy, (3) Eastern Cooperative Oncology Group performance status (ECOG PS) 0–2, and (4) adequate organ function. The following patients were excluded: (1) those that received adjuvant chemotherapy and (2) had other active cancers. We divided the patients into three groups according to the dates of approval of novel anticancer drugs that improve survival at the initiation of first‐line treatment; patients who initiated first‐line treatment from Jan 2005 to Dec 2006 were included in cohort A (only cytotoxic drugs available), patients from Jan 2007 to Dec 2011 were included in cohort B (molecular targeting drugs available), and those who began treatment from Jan 2012 to Sep 2016 were included in cohort C (regorafenib or FTD/TPI as a late‐line treatment). In Japan, bevacizumab was approved in 2007, cetuximab in 2008, panitumumab in 2010, regorafenib in 2012, and FTD/TPI in 2014. As for *RAS* testing, *KRAS* testing was approved in 2010 (late cohort B) and all *RAS* testing began in 2015 (late cohort C). The tumor locations were defined as the right side of the colon from the cecum to the splenic flexure and the left side of the colon from the splenic flexure to the rectum. All data were retrospectively collected from medical records, and the cutoff date was December 31, 2019.

The objective of this study is to investigate the impact of drug availability on OS since the initiation of first‐line treatment between cohorts A, B, and C. In addition, we evaluated the impact of primary tumor location, the number of drugs administered, and conversion surgery on OS, as well as the proportion of drugs administered.

All procedures were performed in accordance with institutional and national standards on human experimentation, as confirmed by the ethics committee of Shizuoka Cancer Center (IRB number 2161), Aichi Cancer Center (IRB number 2019–1‐201), and Hokkaido University Hospital (IRB number 019–0176) as well as with the Declaration of Helsinki of 1964 and later versions.

### 2.1 Statistical analyses

OS was defined as the duration between start date of first‐line treatment and date of death, date of last follow‐up, or the cutoff date of this study (December 31, 2019). Survival rates were estimated using the Kaplan–Meier method and were compared using the log‐rank test. To simultaneously evaluate the effect of several factors on survival, multivariable Cox regression analyses were performed. The association between categorical parameters was analyzed with a Chi‐squared or Fisher's exact test. Continuous variables were analyzed with Student's *t* test. The analyses were performed using the statistical software R (R Foundation for Statistical Computing, v. 4.0.3). All reported *p*‐values were two‐sided, and a *p*‐value <0.05 was considered statistically significant.

## 3 RESULTS

### 3.1 Patient characteristics

Between January 2005 and November 2016, 1409 consecutive patients initiated systemic first‐line treatment for mCRC. The patient characteristics were similar among the three groups, excluding age and resection of primary tumor, as shown in Table 1. The median age (interquartile range, IQR) was 62.0 (54.0–69.0) years in cohort A, 63.0 (56.0–70.0) years in cohort B, and 65.0 (55.0–72.0) years in cohort C. Resection of the primary tumor was performed significantly more often in cohort C compared with cohorts A and B. Many patients in cohorts A and B had unknown *KRAS* status since testing for mutations in this gene was approved in 2010 in Japan.

**TABLE 1:** Patients' characteristics

	Cohort A (*N* = 165)	cohort B (*N* = 622)	cohort C (*N* = 622)	*p* value
Age (years)				
Median (IQR)	62 (54, 69)	63 (56, 70)	65 (55, 72)	0.019
Sex				
Male	97 (58.8%)	388 (62.4%)	354 (56.9%)	0.142
Female	68 (41.2%)	234 (37.6%)	268 (43.1%)	
ECOG PS				
0–1	151 (91.5%)	571 (91.8%)	579 (93.1%)	0.636
2	14 (8.5%)	51 (8.2%)	43 (6.9%)	
Location				
Right	43 (26.1%)	181 (29.3%)	184 (29.7%)	0.654
Left	122 (73.9%)	437 (70.7%)	436 (70.3%)	
Unknown	0	4	2	
Resection of the primary tumor				
+	100 (60.6%)	386 (62.1%)	335 (53.9%)	0.011
−	65 (39.4%)	236 (37.9%)	287 (46.1%)	
Pathology				
wel/mod	125 (89.9%)	511 (87.2%)	533 (88.0%)	0.671
por/muc	14 (10.1%)	75 (12.8%)	73 (12.0%)	
Unknown	26	36	16	
KRAS status				
Wild	15 (71.4%)	265 (61.8%)	354 (59.1%)	0.401
Mutation	6 (28.6%)	164 (38.2%)	245 (40.9%)	
Unknown	144	193	23	
Liver met.				
−	61 (37.0%)	191 (30.7%)	222 (35.7%)	0.111
+	104 (63.0%)	431 (69.3%)	400 (64.3%)	
Peritoneum met.				
−	134 (81.2%)	470 (75.6%)	450 (72.3%)	0.056
+	31 (18.8%)	152 (24.4%)	172 (27.7%)	
Number of metastatic sites				
<2	60 (36.4%)	240 (38.6%)	261 (42.0%)	0.3
≥2	105 (63.6%)	382 (61.4%)	361 (58.0%)	
WBC				
<10,000/μL	144 (87.3%)	541 (87.0%)	544 (87.5%)	0.968
≥10,000/μL	21 (12.7%)	81 (13.0%)	78 (12.5%)	
ALP				
<300 IU/L	74 (45.7%)	254 (41.2%)	301 (48.4%)	0.037
≥300 IU/L	88 (54.3%)	363 (58.8%)	321 (51.6%)	
Unknown	3	5	0	
LDH				
<400 U/L	111 (67.3%)	418 (67.3%)	445 (72.0%)	0.165
≥400 U/L	54 (32.7%)	203 (32.7%)	173 (28.0%)	
Unknown	0	1	4	

### 3.2 Treatment

Drugs administered throughout the treatment course are shown in Table 2. All patients received fluoropyrimidines, and oxaliplatin was more often used than irinotecan in all cohorts. More patients in cohort C received anti‐angiogenesis drugs (84.1%) compared with cohorts A (21.2%) and B (72.5%). The proportion of patients who were treated with anti‐EGFR therapy was comparable in cohorts B (33.0%) and C (37.9%). More patients in cohort C (49.2%) received regorafenib or FTD/TPI than cohort B (17.1%). The percentage of patients who could receive regorafenib or FTD/TPI was 1.7% in cohort A, 17.1% in cohort B, and 49.2% in cohort C; those who received both regorafenib and FTD/TPI were 0%, 3.1%, and 16.0%, respectively (Table S1).

**TABLE 2:** Contents of treatment during whole treatment period

Treatment	Cohort A (*N* = 165)	Cohort B (*N* = 622)	Cohort C (*N* = 622)	*p*‐value
Chemotherapy				
Fluoropyrimidine	165 (100%)	622 (100%)	622 (100%)	
Oxaliplatin	147 (89.1%)	571 (91.8%)	536 (86.2%)	0.007
Irinotecan	124 (75.2%)	432 (69.5%)	425 (68.3%)	0.236
Anti‐angiogenesis	35 (21.2%)	451 (72.5%)	523 (84.1%)	<0.001
Anti‐EGFR therapy	24 (14.5%)	205 (33.0%)	236 (37.9%)	<0.001
Regorafenib	1 (0.6%)	42 (6.8%)	111 (17.8%)	<0.001
FTD/TPI	1 (0.6%)	43 (6.9%)	168 (27.0%)	<0.001
Conversion surgery	6 (3.6%)	62 (10.0%)	53 (8.5%)	0.036

Abbreviations: EGFR, epidermal growth factor receptor; FTD/TPI, trifluridine/tipiracil.

### 3.3 Overall survival

**FIGURE 1:**
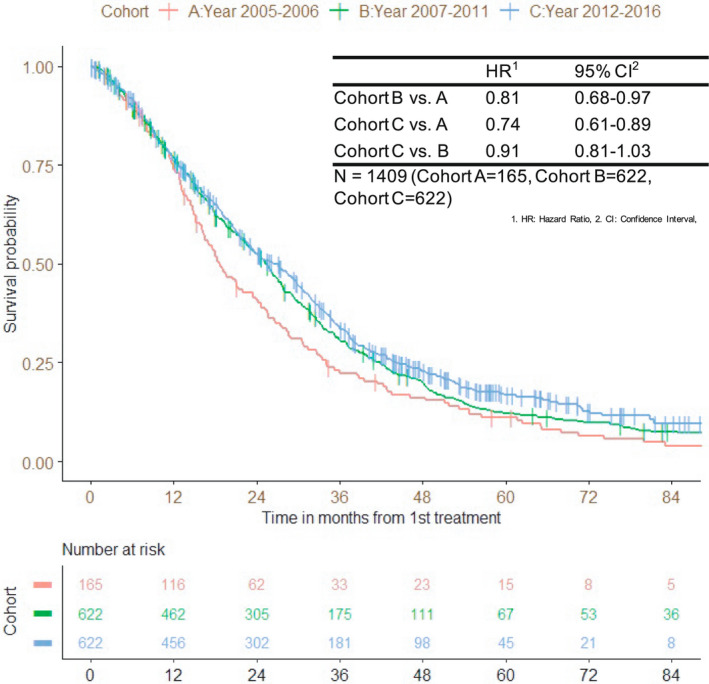
Overall survival according to the date of the initiation of first‐line treatment

While the median follow‐up time (IQR) was 17.7 (11.2–32.7) months in cohort A, 23.2 (11.6–38.9) months in cohort B, and 23.3 (11.6–38.2) months in cohort C, the median survival time (MST) was 18.6 (95% CI 16.4–23.5) months, 25.4 (95% CI 23.2–27.1) months, and 26.4 (95% CI 23.3–29.5) months, respectively (Figure 1). The 2‐year OS rate in each cohort was 40.8%, 52.5%, and 52.5%, and the 3‐year OS rate was 22.4%, 30.9%, and 34.0%, respectively. The hazard ratio (HR) for cohort B versus A was 0.81 (95% CI 0.68–0.97), for cohort C versus A was 0.74 (95% CI 0.61–0.89), and for cohort C versus B was 0.91 (95% CI 0.81–1.03). The adjusted HR for cohort B versus A was 0.78 (95% CI 0.65–0.94), for cohort C versus A was 0.74 (95% CI 0.62–0.90), and for cohort C versus B was 0.95 (95% CI 0.84–1.07).

Drug availability gradually increased from cohorts A to B and C: the median number of administered drugs (range) was 3 (1, 2, 3, 4, 5) in cohort A, 4 (1, 2, 3, 4, 5, 6, 7) in cohort B, and 4 (1, 2, 3, 4, 5, 6, 7) in cohort C (Figure 3A). The proportion of patients treated with ≤3 drugs decreased from 71.5% in cohort A to 32.6% in cohort C, while, on the other hand, treatment with ≥6 drugs increased from 6.6% in cohort B to 19.7% in cohort C. The increase of drug availability extended the MST from 15.5 months in patients treated with ≤3 drugs to 36.0–37.3 months in patients treated with 6–7 drugs (Figure 3B).

**FIGURE 2:**
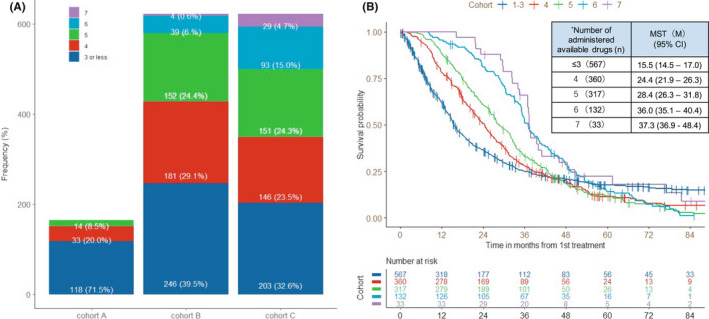
(A) The frequency of administered drugs and overall survival (OS). (B) OS according to the number of administered available drugs*

As for the primary tumor location, the MST of left‐sided tumors was significantly longer than for right‐sided tumors (27.6 months vs. 18.3 months; HR 0.71; 95% CI 0.62–0.80) (Figure 2A). For patients with left‐sided tumors, the prognosis tended to be better with period: the HR for cohort B versus A was 0.77 (95% CI 0.62–0.95), for cohort C versus A was 0.68 (95% CI 0.55–0.85), and for cohort C versus B was 0.89 (95% CI 0.77–1.04) (Figure 2B). On the other hand, for patients with right‐sided tumors, the prognosis of cohort B tended to be better than that of cohort A and the prognosis of cohort B was comparable with cohort C: the HR for cohort B versus A was 0.89 (95% CI 0.63–1.26), and for cohort C versus B was 0.95 (95% CI 0.76–1.18; Figure 2C). There was no difference in the number of drugs administered between the left‐ and right‐sided (Table S2).

**FIGURE 3:**
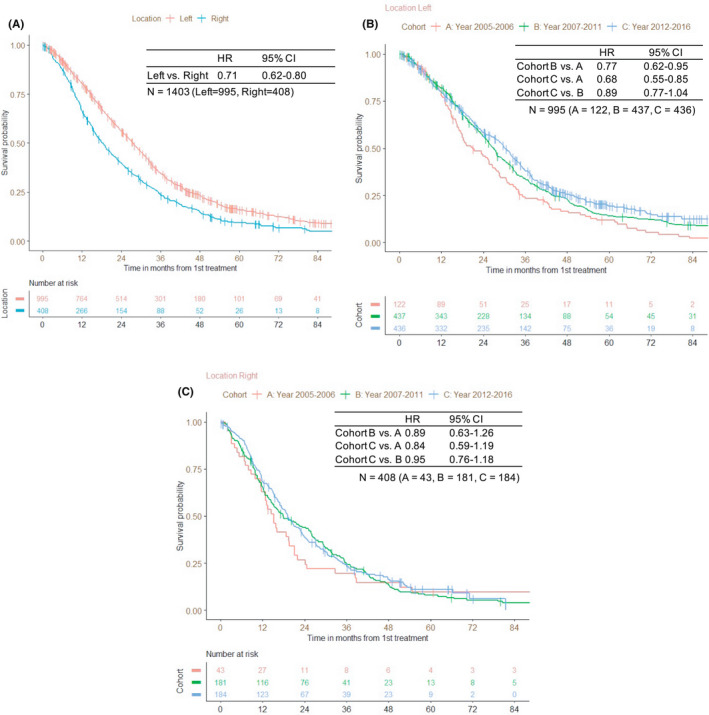
(A) Overall survival (OS) from the date of the initiation of first‐line treatment (tumor location). (B) OS of left‐sided tumors from the initiation of first‐line treatment. (C) OS of right‐sided tumors from the initiation of first‐line treatment. *Re‐challenge or investigational drugs were not included. Cetuximab and panitumumab counts it with one drug as anti‐EGFR antibody. Bevacizumab, ramucirumab, and ziv‐aflibercept counts it with one drug as anti‐angiogenesis drug

Among the patient factors, a Cox proportional hazard model revealed that ECOG PS 2–3, right‐sided tumors, ≥2 metastatic sites, a white blood cell count ≥10,000/μL, ALP ≥ 300 IU/L, and LDH ≥ 400 IU/L were independent poor prognostic factors (Table 3). After adjusting for these prognostic factors, the adjusted HR for cohort B versus A was 0.78 (95% CI 0.65–0.94), for cohort C versus A was 0.74 (95% CI 0.62–0.90), and for cohort C versus B was 0.95 (95% CI 0.84–1.07) (Figure 1).

**TABLE 3:** Multivariable analysis for total overall survival (Cox proportional hazard model^a^)

Variable	Levels	Multivariable analysis	
		HR	95% CI
ECOG PS [ref. 0 or 1]	2	2.33	1.88–2.90
Tumor location [ref. Left]	Right	1.36	1.20–1.54
Number of metastatic sites [ref. 0 or 1]	≥2	1.46	1.29–1.65
WBC [ref. < 10,000/μL]	≥10,000/μL	1.58	1.33–1.89
ALP [ref. < 300 IU/L]	≥300 IU/L	1.32	1.16–1.51
LDH [ref. < 400 U/L]	≥400 U/L	1.40	1.21–1.61

Abbreviations: CI, confidence interval; HR, hazard ratio.

^a^ Adjusted sex and age.

### 3.4 Conversion surgery

The proportion of patients who underwent conversion surgery in cohorts B and C were higher than that in cohort A, and that in cohort B was comparable with cohort C (3.6% in cohort A, 10.0% in cohort B, and 8.5% in cohort C; Table 2). The MST of patients who underwent conversion surgery was significantly longer than those who did not (62.0 months vs. 22.6 months; HR 0.30; 95% CI 0.23–0.38) (Figure S1). The conversion surgery rate was approximately 5–10% every year (Figure S2). The HR for death was comparable regardless of cohort, at 0.39 (95% CI 0.16–0.94) in cohort A, 0.33 (95% CI 0.24–0.45) in cohort B, and 0.25 (95% CI 0.16–0.38) in cohort C (Figure S3).

## 4 DISCUSSION

This study demonstrates that patients with mCRC who initiated first‐line therapy treatment after the date when molecular targeting agents became available (cohort B) and when late‐line treatment with regorafenib or FTD/TPI (cohort C) were available had significantly longer OS than previously, when only cytotoxic chemotherapy was available (cohort A), after adjustment for prognostic factors. Cohort C showed a trend toward a longer survival time compared to cohort B, but the difference was not significant.

The strength of this study is that these results reflect the development of survival benefits in chemotherapy treatments for mCRC in clinical practice using a large number of cases at oncology facilities. Compared with other countries, both FTD/TPI and regorafenib were used more frequently in Japanese clinical practice because the both drugs were approved and could be administered as sequential therapies. Hence, the results of the present study could reflect close to the true survival benefit of development of chemotherapy for mCRC.

The number of drugs administered increased over time. All the approved drugs are well established in clinical practice. As for molecular targeting agents, the proportion of anti‐angiogenesis agents used increased with each cohort, but the use of anti‐EGFR antibodies was similar between cohorts B and C. Anti‐EGFR antibodies were initially intended for patients with positive EGFR expression by immunohistochemistry. However, their use was later narrowed to *KRAS* wild‐type patients, all *RAS* wild‐type patients, and *RAS* wild‐type patients with left‐sided tumors, who were more likely to receive a survival benefit from the drug. Therefore, the proportion of anti‐EGFR therapy administered was likely to be comparable between cohorts B and C. In cohort C, FTD/TPI or regorafenib could be administered as late‐line treatment at the initiation of first‐line treatment; however, the proportion of FTD/TPI or regorafenib was low. This is probably because of the short observation period in cohort C and the inability to move to late‐line treatment due to poor disease status.

The survival impact of combination therapy with molecular targeting agents in first‐ and second‐line treatment was far greater than that of late‐line treatment with FTD/TPI or regorafenib, and the survival impact of late‐line treatment on whole treatment course was not clear in this study. Possible reasons are as follows: first, as described above, the proportion of FTD/TPI or regorafenib administered in cohort C was lower than that of the molecular‐targeted drugs in cohort B. Second, a learning curve is also thought to affect survival benefit. In the early cases of cohort C, physicians may not have been sufficiently familiar with the management of FTD/TPI and regorafenib, and the effects of these drugs may not have been fully realized. In addition, these cases included patients who were heavily treated, and it is possible that FTD/TPI and regorafenib were administered in cases with more advanced disease states.

Consistent with a previous report,^20^ the OS of patients who underwent conversion surgery was significantly longer than those who did not. Although the proportion of patients who underwent conversion surgery in cohorts B and C was higher than that of cohort A, the impact of conversion surgery on OS was comparable in each cohort.

As for tumor location, the increase of drug availability contributed to prolonging the OS of patients with left‐sided tumors; however, the effect was only slight for patients with right‐sided tumors. There was no significant difference in the number of drugs administered between the left‐sided and right‐sided tumor groups. Right‐sided tumors are known to have worse prognosis compared with left‐sided tumors,^21^ and the survival benefit of each regimen may be poor. Although some studies have demonstrated that the OS of regorafenib or FTD/TPI in right‐sided tumors was similar with that of left‐sided tumors,^22^, ^23^ the survival benefit was poor. Hence, this may reflect a difference in the efficacy of the initial treatment, especially in the antitumor effects of anti‐EGFR therapy. The poor survival benefit of anti‐EGFR therapy in the right‐sided tumor group may result in a small prognostic benefit, and as a result, the antitumor effect of the increase in the number of drugs administered on the OS of patients with right‐sided tumors was poor.

This study has some limitations. First, because of the nature of this retrospective study, a bias in the background of the patients is inevitable. Although age and resection of the primary tumor were significantly different between cohorts A, B, and C, even after adjusting for prognostic factors, the adjusted HR for OS tended to be the same as before adjustment. Hence, this supports the robustness of the results of this study. Second, patients who received adjuvant chemotherapy after curative resection were excluded. Usually, recurrence within 6 months of completion of adjuvant chemotherapy is considered refractory to the drug. In such cases, the date of initiation of first‐line treatment can be ambiguous. Therefore, only patients with synchronous metastases were included in this study, and the prognosis in this study tended to be worse than that of previous reports.

In conclusion, the increase in the administration drugs prolonged OS, and in particular, the MST of patients treated with all available drugs including late‐line treatment with regorafenib and/or FTD/TPI was over 35 months. The development of chemotherapy including late‐line treatment therefore could contribute to improvements in the prognosis of mCRC patients.

## CONFLICT OF INTEREST

The authors declare the following conflicts of interest. Takeshi Kawakami received honoraria from Bayer, Taiho Pharmaceutical, Takeda, Ono Pharmaceutical, Bristol‐Myers Squibb. Toshiki Masuishi received personal fees from Takeda, Chugai, Merck Bio Pharma, Taiho, Bayer, Lilly Japan, Yakult Honsha, and Sanofi. Satoshi Yuki received honoraria from Chugai Pharmaceutical, Eli Lilly, Takeda Pharmaceutical, Bayer, Bristol‐Myers Squibb, Taiho Pharmaceutical, MSD, Ono Pharmaceutical, Medical & Biological Laboratories, Yakult Honsha, Merck Biopharma, and Sanofi. Yoshito Komatsu received grants from Taiho Pharmaceutical and Chugai Pharmaceutical, and honoraria from Eli Lilly Japan, Takeda, Chugai, Daiichi Sankyo, Taiho Pharmaceutical, and Ono Pharmaceutical. Kei Muro received grants from Solasia Pharma, grants from Merck Serono, grants from Daiichi Sankyo, grants from Parexel International, grants from Pfizer, grants from MSD, grants and personal fees from Amgen, grants and personal fees from ONO Pharmaceutical CO., LTD., grants and personal fees from Sanofi, grants and personal fees from Taiho, personal fees from AstraZeneca, personal fees from Chugai, personal fees from Takeda, personal fees from Eli Lilly, personal fees from Bristol‐Myers Squibb, personal fees from Bayer, outside the submitted work. Kentaro Yamazaki received honoraria from Chugai Pharma, Daiichi Sankyo, Yakult Honsha, Takeda, Bayer, Merck Serono, Taiho Pharmaceutical, Lilly, Sanofi, Ono Pharmaceutical, MSD, and Bristol‐Meyers Squibb, also received research funding from Taiho Pharmaceutical.

## AUTHOR CONTRIBUTIONS

*Conceptualization, Methodology, Investigation, Writing – Original Draft, Project Administration, and Supervision*: *Takeshi Kawakami. Investigation, Writing – Review & Editing*: Toshiki Masuishi, Yasuyuki Kawamoto, Kyoko Kato, Ryosuke Kumanishi, Kentaro Sawada, Satoshi Yuki, Yoshito Komatsu, Kei Muro, Kunihiro Fushiki, Hiromichi Shirasu, and Kentaro Yamazaki. *Formal analysis, Data Curation, Writing – Review & Editing*: Hirofumi Go. *Writing – Review & Editing*: Kouji Yamamoto.

## ETHICAL CONSIDERATION

All protocol and procedures were approved by the ethics committee of Shizuoka Cancer Center (IRB number 2161), Aichi Cancer Center (IRB number 2019–1‐201), and Hokkaido University Hospital (IRB number 019–0176).

## Supporting information


Figure S1

Figure S2

Figure S3A

Figure S3B

Figure S3C

DataS 1

Tables S1‐S2

## Data Availability

The data that support the findings of this study are available from the corresponding author upon reasonable request.

## References

[1] The Global Cancer Observatory . https://gcoiarcfr/today/data/factsheets/cancers/10_8_9‐Colorectum‐fact‐sheetpdf

[2] Statistics of Cancer Center for information service. National Cancer Center. 2021. https://ganjohojp/reg_stat/statistics/stat/summaryhtml

[3] Venook A . Critical evaluation of current treatments in metastatic colorectal cancer. Oncologist. 2005;10:250‐261. doi:10.1634/theoncologist.10-4-250 15821245

[4] Machover D , Goldschmidt E , Chollet P , et al. Treatment of advanced colorectal and gastric adenocarcinomas with 5‐fluorouracil and high‐dose folinic acid. J Clin Oncol. 1986;4:685‐696. doi:10.1200/JCO.1986.4.5.685 3517242

[5] Petrelli N , Douglass HO Jr , Herrera L , et al. The modulation of fluorouracil with leucovorin in metastatic colorectal carcinoma: a prospective randomized phase III trial. Gastrointestinal Tumor Study Group. J Clin Oncol. 1989;7:1419‐1426. doi:10.1200/JCO.1989.7.10.1419 2674331

[6] O'Connell MJ . A phase III trial of 5‐fluorouracil and leucovorin in the treatment of advanced colorectal cancer. A Mayo Clinic/North Central Cancer Treatment Group study. Cancer. 1989;63(Supplement):1026‐1030. doi:10.1002/1097-0142(19890315)63:6+<1026::aid-cncr2820631307>3.0.co;2-r 2465076

[7] Saltz LB , Cox JV , Blanke C , et al. Irinotecan plus fluorouracil and leucovorin for metastatic colorectal cancer. Irinotecan Study Group. N Engl J Med. 2000;343:905‐914. doi:10.1056/NEJM200009283431302 11006366

[8] Douillard JY , Cunningham D , Roth AD , et al. Irinotecan combined with fluorouracil compared with fluorouracil alone as first‐line treatment for metastatic colorectal cancer: a multicentre randomised trial. Lancet. 2000;355:1041‐1047. doi:10.1016/s0140-6736(00)02034-1 10744089

[9] Goldberg RM , Sargent DJ , Morton RF , et al. A randomized controlled trial of fluorouracil plus leucovorin, irinotecan, and oxaliplatin combinations in patients with previously untreated metastatic colorectal cancer. J Clin Oncol. 2004;22:23‐30. doi:10.1200/JCO.2004.09.046 14665611

[10] Saltz LB , Clarke S , Díaz‐Rubio E , et al. Bevacizumab in combination with oxaliplatin‐based chemotherapy as first‐line therapy in metastatic colorectal cancer: a randomized phase III study. J Clin Oncol. 2008;26:2013‐2019. doi:10.1200/JCO.2007.14.9930 18421054

[11] Hurwitz H , Fehrenbacher L , Novotny W , et al. Bevacizumab plus irinotecan, fluorouracil, and leucovorin for metastatic colorectal cancer. N Engl J Med. 2004;350:2335‐2342. doi:10.1056/NEJMoa032691 15175435

[12] Van Cutsem E , Köhne CH , Hitre E , et al. Cetuximab and chemotherapy as initial treatment for metastatic colorectal cancer. N Engl J Med. 2009;360:1408‐1417. doi:10.1056/NEJMoa0805019 19339720

[13] Heinemann V , von Weikersthal LF , Decker T , et al. FOLFIRI plus cetuximab versus FOLFIRI plus bevacizumab as first‐line treatment for patients with metastatic colorectal cancer (FIRE‐3): a randomised, open‐label, phase 3 trial. Lancet Oncol. 2014;15:1065‐1075. doi:10.1016/S1470-2045(14)70330-4 25088940

[14] Douillard JY , Siena S , Cassidy J , et al. Final results from PRIME: randomized phase III study of panitumumab with FOLFOX4 for first‐line treatment of metastatic colorectal cancer. Ann Oncol. 2014;25:1346‐1355. doi:10.1093/annonc/mdu141 24718886

[15] Mayer RJ , Van Cutsem E , Falcone A , et al. Randomized trial of TAS‐102 for refractory metastatic colorectal cancer. N Engl J Med. 2015;372:1909‐1919. doi:10.1056/NEJMoa1414325 25970050

[16] Grothey A , Van Cutsem E , Sobrero A , et al. Regorafenib monotherapy for previously treated metastatic colorectal cancer (CORRECT): an international, multicentre, randomised, placebo‐controlled, phase 3 trial. Lancet. 2013;381:303‐312. doi:10.1016/S0140-6736(12)61900-X 23177514

[17] Yamada Y , Denda T , Gamoh M , et al. S‐1 and irinotecan plus bevacizumab versus mFOLFOX6 or CapeOX plus bevacizumab as first‐line treatment in patients with metastatic colorectal cancer (TRICOLORE): a randomized, open‐label, phase III, noninferiority trial. Ann Oncol. 2018;29:624‐631. doi:10.1093/annonc/mdx816 29293874 PMC5889030

[18] Loupakis F , Cremolini C , Masi G , et al. Initial therapy with FOLFOXIRI and bevacizumab for metastatic colorectal cancer. N Engl J Med. 2014;371:1609‐1618. doi:10.1056/NEJMoa1403108 25337750

[19] Schwartzberg LS , Rivera F , Karthaus M , et al. PEAK: a randomized, multicenter phase II study of panitumumab plus modified fluorouracil, leucovorin, and oxaliplatin (mFOLFOX6) or bevacizumab plus mFOLFOX6 in patients with previously untreated, unresectable, wild‐type KRAS exon 2 metastatic colorectal cancer. J Clin Oncol. 2014;32:2240‐2247. doi:10.1200/JCO.2013.53.2473 24687833

[20] Adam R , Wicherts DA , de Haas RJ , et al. Patients with initially unresectable colorectal liver metastases: is there a possibility of cure? J Clin Oncol. 2009;27:1829‐1835. doi:10.1200/JCO.2008.19.9273 19273699

[21] Arnold D , Lueza B , Douillard JY , et al. Prognostic and predictive value of primary tumour side in patients with RAS wild‐type metastatic colorectal cancer treated with chemotherapy and EGFR directed antibodies in six randomized trials. Ann Oncol. 2017;28:1713‐1729. doi:10.1093/annonc/mdx175 28407110 PMC6246616

[22] Nagaoka T , Wakatsuki T , Shinozaki E , et al. Prognostic impact of primary tumor location in patients with metastatic colorectal cancer (mCRC) at the salvage lines. J Clin Oncol. 2017;35:741.

[23] Michel Ducreux LNP , Leopold Ö , Francesca B , et al. Outcomes by tumor location in patients with metastatic colorectal cancer (mCRC) treated with regorafenib (REG): final analysis from the prospective, observational CORRELATE study. J Clin Oncol. 2019;37:539.

