# Supplementary figures and images for: The survival benefit of increasing the number of active drugs for metastatic colorectal cancer: A multicenter retrospective study
Source: Cancer Med. 2022 Feb 19;11(11):2184–92. doi: 10.1002/cam4.4599 (PMC9160807; doi:10.1002/cam4.4599)

Cohort + no conversion surgery + conversion surgery

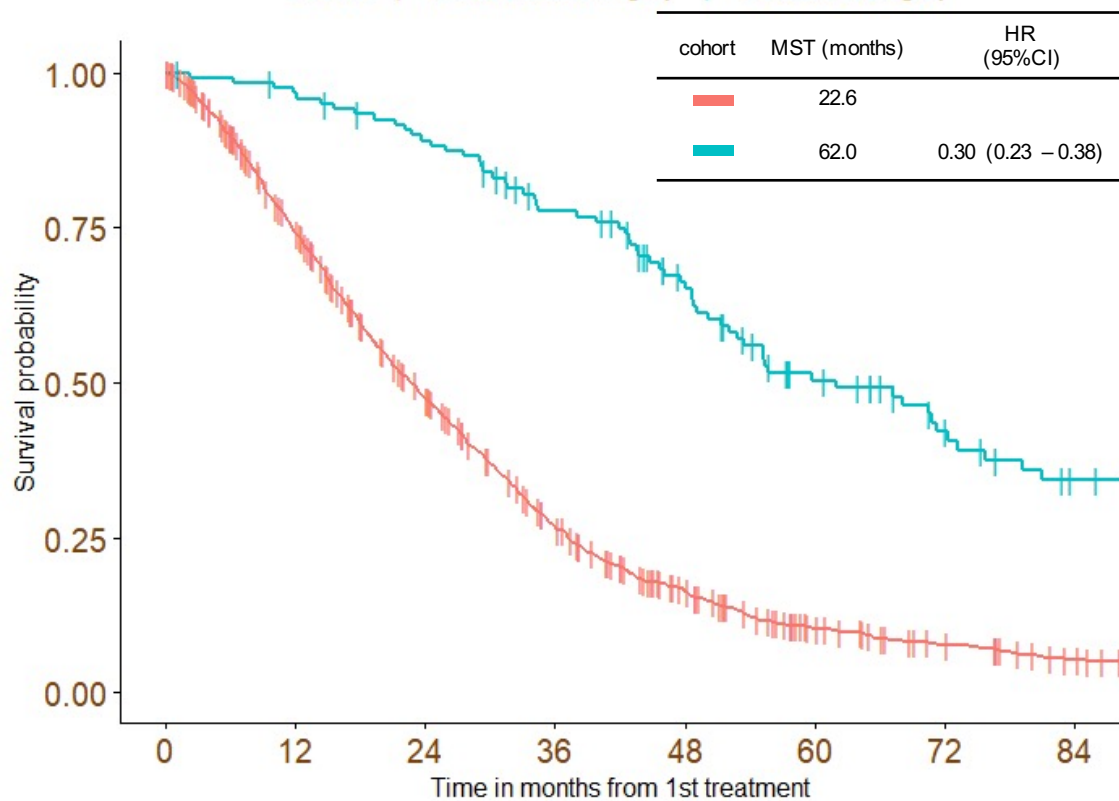

Number at risk

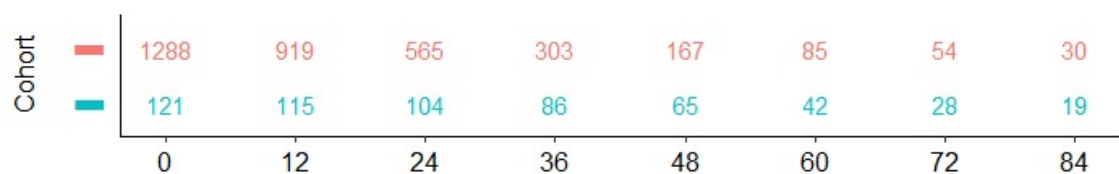

Supplement: Supplementary file 1 — Figure S1 [file CAM4-11-2184-s007.pdf]

Undergoing conversion surgery

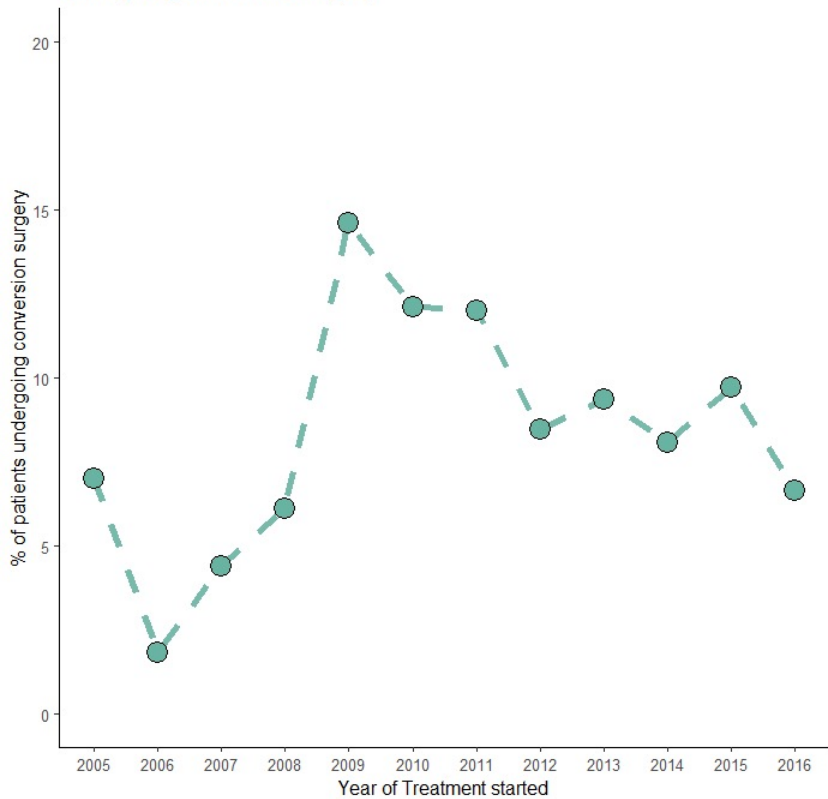

Supplement: Supplementary file 2 — Figure S2 [file CAM4-11-2184-s001.pdf]

# Cohort A

Cohort + no conversion surgery + conversion surgery

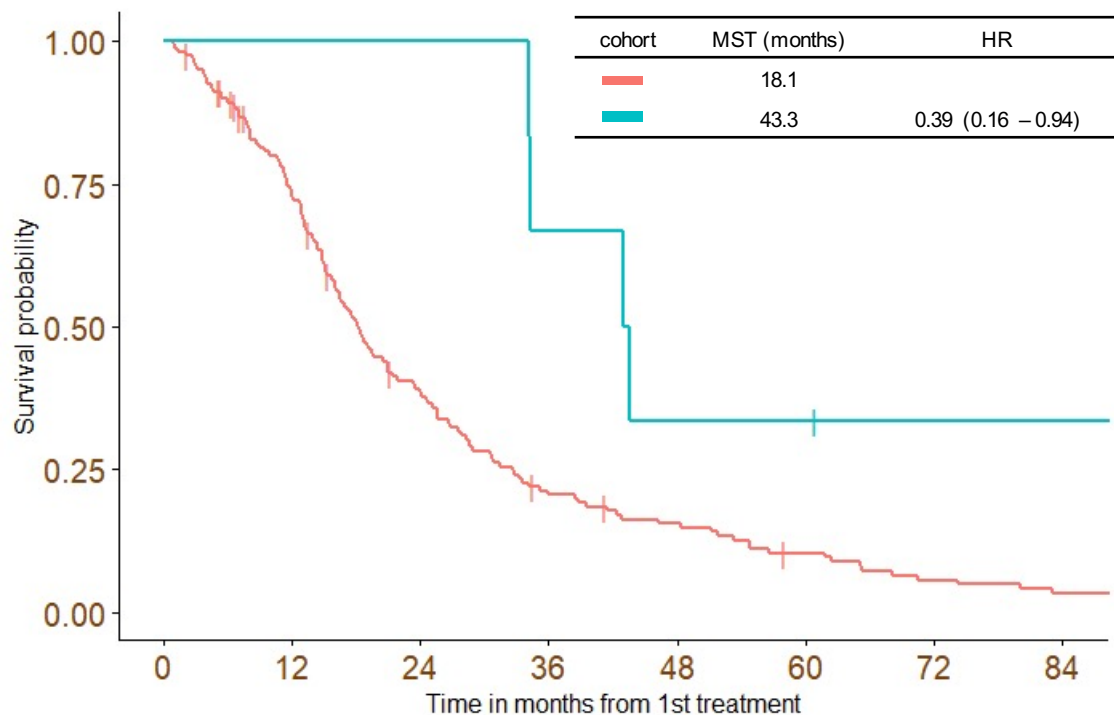

Number at risk

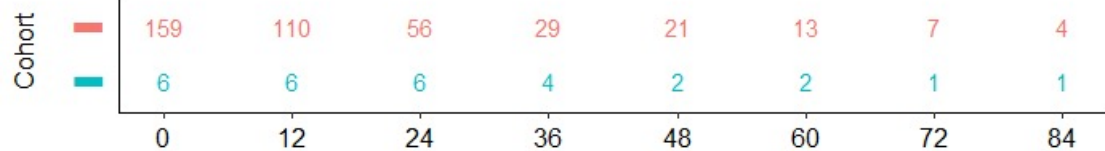

Supplement: Supplementary file 3 — Figure S3A [file CAM4-11-2184-s006.pdf]

# Cohort B

Cohort + no conversion surgery + conversion surgery

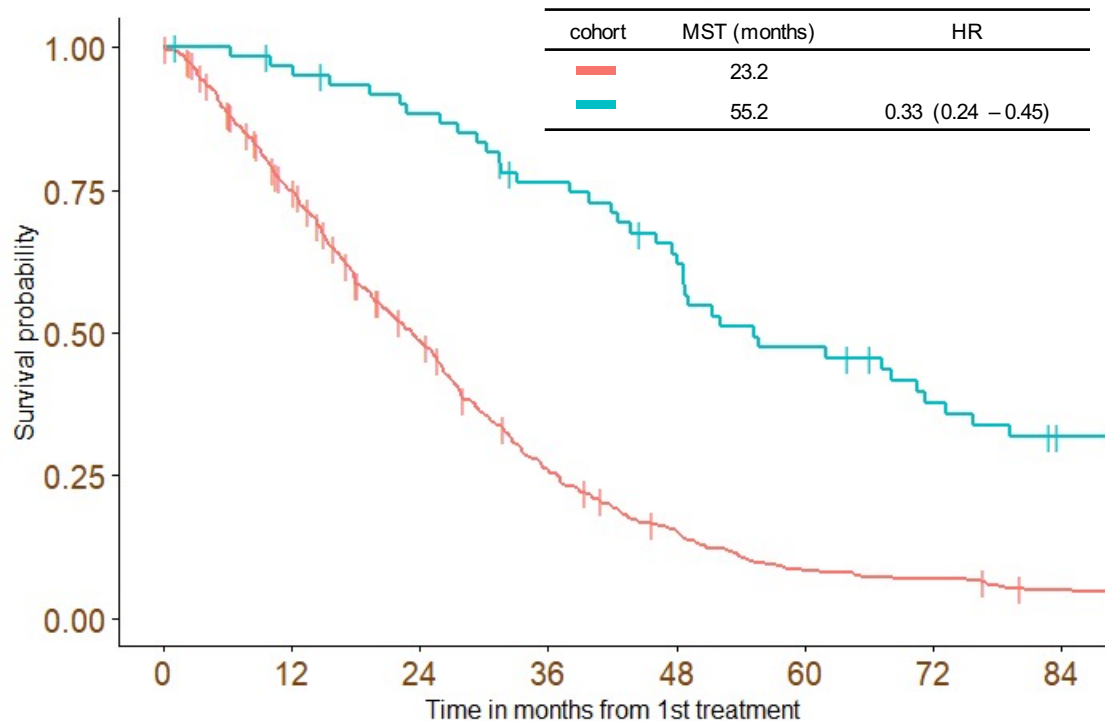

Number at risk

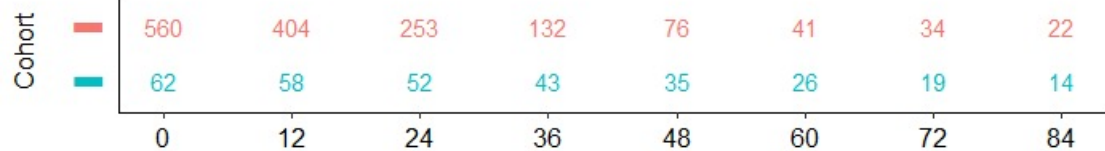

Supplement: Supplementary file 4 — Figure S3B [file CAM4-11-2184-s005.pdf]

# Cohort C

Cohort + no conversion surgery + conversion surgery

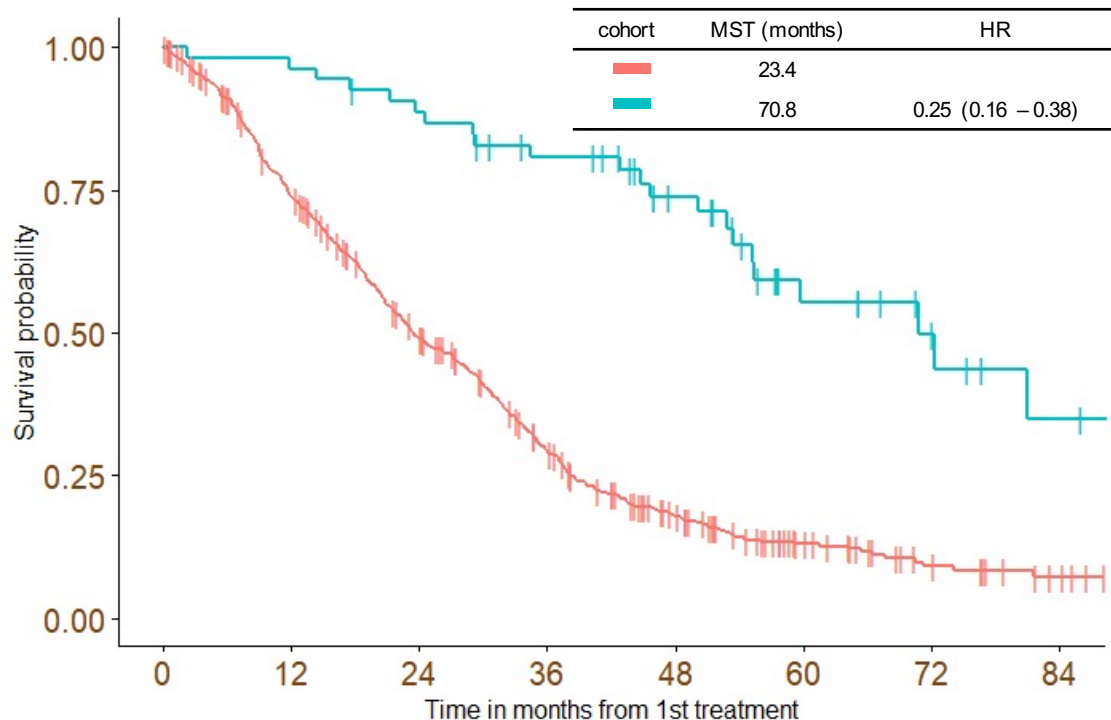

Number at risk

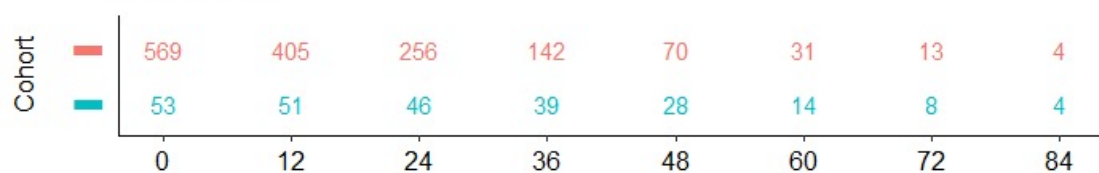

Supplement: Supplementary file 5 — Figure S3C [file CAM4-11-2184-s004.pdf]
