# Supplementary material for: The survival benefit of increasing the number of active drugs for metastatic colorectal cancer: A multicenter retrospective study
Source: Cancer Med. 2022 Feb 19;11(11):2184–92. doi: 10.1002/cam4.4599 (PMC9160807; doi:10.1002/cam4.4599)
Supplement: Supplementary file 6 — DataS 1 [file CAM4-11-2184-s002.docx]

Fig. S1. OS according to conversion surgery or not

Fig. S2. Transition of conversion surgery rate

Fig. S3. Survival impact of conversion surgery on OS of each cohort

(A) cohort A (B) cohort B (C) cohort C
