# Supplementary material for: The survival benefit of increasing the number of active drugs for metastatic colorectal cancer: A multicenter retrospective study
Source: Cancer Med. 2022 Feb 19;11(11):2184–92. doi: 10.1002/cam4.4599 (PMC9160807; doi:10.1002/cam4.4599)
Supplement: Supplementary file 7 — Tables S1‐S2 [file CAM4-11-2184-s003.docx]

Table S1. The administration of late-line treatment in each cohort.

Table S2. The number of administered drugs according to tumor sidedness

Table S1. The administration of late-line treatment in each cohort.

|  | cohort A (N=120) | cohort B (N=420) | cohort C (N=425) |
| --- | --- | --- | --- |
| Non-FTD/TPI / REGO | 118 (98.3%) | 348 (82.9%) | 216 (50.8%) |
| REGO only | 1 (0.8%) | 29 (6.9%) | 42 (9.9%) |
| FTD/TPI only | 1 (0.8%) | 30 (7.1%) | 99 (23.3%) |
| REGO or FTD/TPI | 2 (1.7%) | 72 (17.1%) | 209 (49.2%) |
| REGO and FTD/TPI | 0 (0.0%) | 13 (3.1%) | 68 (16.0%) |
| N = 965（Patients who received second and subsequent treatments） | | | |

Table S2. The number of administered drugs according to tumor sidedness

| The number of administered drugs | Left-sided (N=995) | | Right-sided(N=408) | | *P* value |
| --- | --- | --- | --- | --- | --- |
|  | N | % | N | % |  |
| 1 | 22 | 2.2 | 13 | 3.2 | 0.249 |
| 2 | 126 | 12.7 | 48 | 11.8 |  |
| 3 | 254 | 25.5 | 100 | 24.5 |  |
| 4 | 244 | 24.5 | 115 | 28.2 |  |
| 5 | 228 | 22.9 | 88 | 21.6 |  |
| 6 | 92 | 9.2 | 40 | 9.8 |  |
| 7 | 29 | 2.9 | 4 | 1.0 |  |
